# Editorial: Advances of brain metastasis in breast cancer

**DOI:** 10.3389/fnhum.2025.1554890

**Published:** 2025-01-22

**Authors:** Wenjie Lv, Adam Brufsky, Takahiro Ochiya

**Affiliations:** ^1^Department of Breast Surgery, Xinhua Hospital Affiliated to Shanghai Jiao Tong University School of Medicine, Shanghai, China; ^2^UPMC Hillman Cancer Center, University of Pittsburgh, Pittsburgh, PA, United States; ^3^Department of Molecular and Cellular Medicine, Tokyo Medical University, Tokyo, Japan

**Keywords:** brain metastasis, breast cancer, blood-brain barrier, therapy resistance, targeted therapy

Breast cancer is the most frequently diagnosed malignancy among women worldwide and a leading cause of cancer-related deaths. Brain metastasis, a severe complication of advanced breast cancer, significantly worsens the prognosis due to the challenges posed by the blood-brain barrier (BBB) and the aggressive nature of metastatic cells (Raghavendra and Ibrahim, 2024). The development of brain metastases from breast cancer involves multiple steps, including the detachment of cancer cells from the primary tumor, invasion into the bloodstream, and colonization of the brain (Ivanova et al., 2023). The BBB, which protects the brain from harmful substances, also limits the effectiveness of many chemotherapeutic agents. Recent studies have identified several molecular pathways and genetic mutations that facilitate the penetration of breast cancer cells through the BBB and their subsequent growth in the brain. Key players include the HER2 (human epidermal growth factor receptor 2) and BRCA (breast cancer gene) mutations, which are associated with more aggressive disease and higher likelihood of brain metastasis (Fan et al., 2023; Kuksis et al., 2021).

This Research Topic aims to highlight the recent advances in the treatments of brain metastasis in breast cancer, including the discovery of new targets or agents, novel methods in pre-clinical or clinical trials, as well as deeper insights focusing on the known agents and treatments.

Understanding the molecular basis of central nervous system metastases is crucial for developing effective treatments. Lipocalin-2 (LCN2), an iron transport protein, is implicated in the progression of breast cancer brain metastasis (BCBM) (Adler et al., 2023). In primary tumors, LCN2 promotes cancer cell proliferation, angiogenesis, and invasion by interacting with matrix metalloproteinase-9 and facilitating epithelial-mesenchymal transition. In the brain microenvironment, LCN2 disrupts the blood-brain barrier and aids tumor seeding by modulating cellular behavior. Zhao et al. reviewed LCN2′s role in BCBM and its potential as a therapeutic target and biomarker, suggesting that targeting LCN2 could improve outcomes for BCBM patients.

Extracellular vesicles (EVs), which are small lipid bilayer vesicles containing biomolecules, play a crucial role in this process by delivering bioactive molecules to recipient cells and regulating signal transduction and protein expression. EVs were confirmed to play a key role in the regulation of the immune microenvironment of brain metastasis and are expected to make advances in immunotherapy and disease diagnosis (Li et al., 2024). Sakamoto et al. reviewed the molecular mechanisms through which EVs promote brain metastasis in breast cancer and discusses the potential of EV-associated molecules as therapeutic targets and early diagnostic markers.

Immune checkpoint inhibitors (ICIs) represent a promising option for patients with BCBM, particularly for those with TNBC that otherwise have very limited non-chemotherapy systemic therapy options (Schlam and Gatti-Mays, 2022). However, it remains essential to investigate the factors influencing the efficacy of ICIs treatment. A study found that high tumor mutation burden (TMB) in metastatic lesions suggests potential benefits from immune checkpoint inhibitors for brain metastasis (Deguchi et al., 2024). Additionally, TREML2 and BTLA are identified as poor prognostic factors, and activated microglia may serve as novel treatment targets. Najjary et al. investigated the increasing incidence of brain metastases in cancer patients, particularly from lung and breast cancers, and the poor prognosis despite advancements in targeted therapies. It explored the molecular differences in brain metastasis between these cancers and identify specific druggable targets. Analyzing 44 tissue samples, the study found significant upregulation of cancer-related genes in primary tumors compared to brain metastases. Key findings include the association of upregulated genes with metabolic stress pathways, immune response regulation, tumor growth, and proliferation. Notably, high expression of immune checkpoints VTCN1 in breast cancer brain metastasis and VISTA, IDO1, NT5E, and HDAC3 in lung adenocarcinoma brain metastasis were identified and validated. This study suggested specific immune checkpoints could be identified as potential druggable targets.

Multiple drugs have been validated through clinical trials to be both safe and effective in the treatment of breast cancer brain metastases. The DESTINY-Breast12 (DB12) study evaluated the efficacy and safety of trastuzumab deruxtecan (T-DXd) in patients with HER2-positive metastatic breast cancer, including those with brain metastases. The median progression-free survival (PFS) for patients with brain metastases was 17.3 months, with a 12-month PFS rate of 61.6%. The intracranial objective response rate (CNS ORR) was 71.7%, indicating strong efficacy in treating brain metastases (Harbeck et al., 2024). When combined with trastuzumab and capecitabine, tucatinib has improved intracranial and extracranial progression-free survival and overall survival in patients with HER2-positive brain metastases after previous trastuzumab-deruxtecan treatment (Frenel et al., 2024). Liu et al. evaluated the efficacy and safety of the anti-angiogenic agent anlotinib in treating triple-negative breast cancer (TNBC) patients with brain metastases who had failed prior therapies. Analyzing 29 patients between October 2019 and April 2024, the study found that the median central nervous system progression-free survival (CNS PFS) was 7.2 months, and the median overall survival (OS) was 10.2 months. The intracranial objective response rate (iORR) was 31.0%, and the intracranial disease control rate (iDCR) was 86.2%. Five patients experienced grade 3–4 adverse events, with bone marrow suppression being the most common. Most adverse events were manageable, and no treatment-related deaths occurred. This study suggested anlotinib offered a promising new treatment for TNBC patients with brain metastases.

In conclusion, the articles compiled in this Research Topic offer a thorough examination of the understanding and treatment of breast cancer brain metastasis. However, significant challenges persist. Ongoing research and clinical trials are crucial to developing more effective therapies and enhancing the quality of life for patients suffering from this devastating condition.
